# Role of MRI in Imaging Assessment of Radiation-Based Treatment of Hepatocellular Carcinoma

**DOI:** 10.3390/cancers18071089

**Published:** 2026-03-27

**Authors:** Liang Meng Loy, Guo Yuan How, Uei Pua, Han Hwee Lawrence Quek, Cher Heng Tan

**Affiliations:** 1 Khoo Teck Puat Hospital, Singapore 768828, Singapore; 2 Tan Tock Seng Hospital, Singapore 308433, Singapore; guoyuan.how@mohh.com.sg (G.Y.H.); pua.uei@nhghealth.com.sg (U.P.); lawrence.quek@nhghealth.com.sg (H.H.L.Q.); 3 Woodlands Hospital, Singapore 737628, Singapore; cher.heng.tan@nhghealth.com.sg; 4 Lee Kong Chian School of Medicine, Nanyang Technological University, Singapore 637511, Singapore

**Keywords:** hepatocellular carcinoma, TARE, transarterial radioembolization, treatment response, senescence

## Abstract

Magnetic Resonance Imaging (MRI) plays a pivotal role in evaluating treatment response following radiation-based therapies for hepatocellular carcinoma (HCC). As radiation modalities such as stereotactic body radiotherapy (SBRT) and transarterial radioembolization (TARE) gain prominence, understanding the underlying mechanisms of radiation-induced cellular senescence is essential for accurate interpretation of imaging. The physiological changes of radiation treatment manifest as altered diffusion characteristics and delayed regression of enhancement and tumor volumes on MRI, challenging conventional response criteria. Herein, functional and temporal imaging biomarkers are necessary. However, current imaging strategies lack standardization and robust validation, underscoring the need for prospective studies to correlate MRI findings with treatment outcomes. This review synthesizes emerging evidence on MRI-based evaluation of radiation-treated HCC, explores the physiological rationale linking senescence to imaging phenotypes, and advocates for optimized imaging protocols and criteria to enhance post-treatment surveillance and therapeutic decision-making.

## Simple Summary

Post-imaging MRI findings after radiation-based treatment of HCC (TARE, SBRT) are different from those after non-radiation-based treatment due to underlying radiation-induced cellular senescence. Following TARE and SBRT, persistent enhancement and delayed tumor shrinkage can confound the application of conventional treatment response criteria. Conventional treatment response assessment guidelines need to be adjusted to take into account the different imaging findings after TARE and SBRT.

## 1. Introduction

Patients with hepatocellular carcinoma (HCC) can be treated in a variety of curative and non-curative ways. Curative treatments include liver transplantation, surgical resection and ablation. Non-curative treatments include transarterial chemoembolization (TACE), transarterial radioembolization (TARE), stereotactic body radiation therapy (SBRT), and systemic chemotherapy and immunotherapy. Non-curative treatment aims to slow tumor progression, alleviate symptoms, or prolong survival [1].

There has been an increasing usage of TARE in HCC treatment in the last decade, with promising outcomes [2]. The efficacy of TARE for the treatment of tumors smaller than 8 cm when other therapies are not feasible has been recognized by the Barcelona Clinic Liver Cancer (BCLC) steering committee [3,4], and TARE has been included in the BCLC algorithm for BCLC 0-A cases [4,5].

Following locoregional treatment (LRT), patients undergo follow-up with regular imaging surveillance to assess treatment response. Therefore, it is important that radiologists can accurately interpret post-LRT imaging to guide subsequent clinical management. However, expected imaging findings after radiation-based treatment (TARE, SBRT) are different from those seen after non-radiation-based treatment, which poses an immense challenge when evaluating post-treatment response using existing treatment response guidelines [1]. The latest update to the Liver Imaging and Reporting Data System Treatment Response Algorithm (LI-RADS TRA) in 2024 accounted for the differences in imaging findings after radiation-based treatment [6]. Although a few studies had investigated the differences in imaging findings after radiation-based treatment, they did not interrogate the underlying physiological mechanisms leading to the different imaging findings following treatment [7]. This narrative review aims to assimilate emerging evidence on MRI-based evaluation of radiation-treated HCC and explain the physiological basis leading to their unconventional post-treatment imaging findings, with an emphasis on radiation-induced cell senescence.

## 2. Search Methodology

A literature search was performed in PubMed and Cochrane library electronic databases from inception until September 2025 using the following keywords: [(“HCC” or “hepatocellular carcinoma”) and (“yttrium” or “Y-90” or “Y90” or “radioembolization” or “radio-embolization” or “radioembolisation” or “radio-embolisation” or “TARE” or “SIRT” or “selective internal radiation therapy” or “senescence”)]. Review papers and primary studies were considered for inclusion. References of included articles were also manually searched to identify additional eligible studies.

## 3. Traditional Methods of Treatment Response Assessment

Traditionally, treatment response of solid tumors by cross-sectional imaging was assessed by size criteria: World Health Organization (WHO) criteria (bi-dimensional), Response Criteria in Solid Tumors (RECIST) criteria (unidimensional), where the size of the entire tumor is measured, regardless of enhancement [8]. HCC-specific classification systems, such as the European Association for the Study of the Liver (EASL) guidelines [9] and modified RECIST (mRECIST) guidelines [10] were subsequently developed to incorporate change in tumor enhancement, rather than size change, to assess treatment response. The linear measurements are limited to enhancing components rather than the whole lesion [1,8].

## 4. Implications of Radiation-Based Treatment on Imaging Findings of Tumors

In 2024, the LI-RADS committee released treatment response guidelines specific to radiation-based treatment options. The guidelines categorize the treated tumor into three possible categories (viable, non-progressing and non-viable) based on the presence or absence of mass-like enhancement. The 2024 guidelines also take into account ancillary features that favour the presence of viable disease, such as mild-moderate T2 signal intensity and diffusion restriction [1,6].

TARE entails intra-arterial targeted delivery of microspheres coated with yttrium-90 (^90^Y), a beta-radiation emitter with a short half-life of 2.67 days. The emitted beta-particles induce destruction of the targeted tumor with a limited penetration depth, thus reducing radiation injury to adjacent hepatic tissue [1]. The mechanism is microembolic; blood flow to the targeted tumor is preserved to maximize radiation damage to the tumor [11]. Therefore, TARE-induced tumor necrosis is largely due to radiation injury [1]. The side effects from TARE are more tolerable than the side effects associated with other hepatic embolization procedures [12], with fewer complications and increased time to progression [11].

Assessment of early TARE treatment response is more complex than that after TACE or ablation [1,13]. The treatment effect following percutaneous ablation and TACE is immediate, whereas radiation-induced treatment changes are typically delayed [14]. As such, post-treatment imaging appearance can be different from that of other LRT methods in terms of size and enhancement characteristics [11].

Treatment response assessment using size criteria is difficult in the early period after TARE. An up to 3-month interval may be required before anatomical changes in size can be observed [1,15,16]. In the early post-treatment period, necrosis, degeneration, and haemorrhage can cause tumors to increase in size (pseudoprogression) even with a positive treatment response [17] and this may persist for months [11,17]. In TARE, the ^90^Y microspheres are trapped in the arteriolar capillaries, thereby subjecting the targeted tumor to a very high dose of radiation. While there is eventual tumor necrosis and size change, changes in tumor morphology are typically delayed [18].

The most important feature that suggests residual or recurrent tumor after TARE is a new or enlarging mass-like enhancement [6]. Although completely treated tumors will eventually become necrotic and non-enhancing, this is not immediate; hence, persistent intratumoral enhancement within the first 3 months does not necessarily indicate treatment failure. It is important not to make a diagnosis of viable tumor just based on the presence of arterial phase hyperenhancement (APHE); rather, growth over time is a better key indicator of viability [1]. In a study by Stocker et al. to evaluate the effectiveness of early post-treatment response (median time of 47 days) based on size and enhancement criteria in predicting long-term response (12 months), the high PPV (>0.9) at early follow-up suggested that the majority of patients without a visible viable tumor at early follow-up MRI had a complete response at 12 months. NPV at early follow-up was low (<0.3), which suggested that a significant number of lesions showed some signs of viable tumor on early follow-up MRI, which subsequently resolved over time. This reflected the delayed treatment response after TARE, with variable residual enhancing areas which may not necessarily indicate treatment failure [7].

In addition, peritumoral APHE is also to be expected after TARE. This could be due to multiple factors, such as edema and perfusion changes. Ancillary features such as T2 signal intensity and diffusion restriction can help to distinguish viable tumor from TARE-related perfusion changes [1]. A thin rim of enhancement is commonly seen around treated tumors, believed to reflect inflammatory reaction and fibrous tissue; this should not be misinterpreted as viable tumor [17,19].

SBRT is the use of an external radiation beam to precisely deliver a high dose of radiation, either as a single dose or divided into three to five fractions (hypofractionated dose) to the tumor [20]. The geometry of the beams enables delivery of high radiation dosage to the tumor with less radiation to the adjacent tissue, using fewer fractions than traditional external beam techniques. This maximizes therapeutic efficacy and reduces the risk of radiation-induced liver disease [1].

Similar to TARE, changes in tumor size and enhancement after SBRT may take months to manifest [20]. Up to 75% HCCs treated with SBRT display persistent APHE 3–6 months after therapy [21,22], and can even persist up to and exceed 1 year [20,23]. In the initial months after SBRT, lesions may even increase in size due to hemorrhage or edema [24]. Most HCCs effectively treated with SBRT exhibit a slow decrease in size; due to the slow change over time, there might not be an appreciable change in size between consecutive examinations [1]. A study by Oldrini et al. reported that although the regression of tumor enhancement over time was variable, diffusion restriction and T2 signal intensities were predictive of treatment response [25].

SBRT may also trigger an inflammatory response in the tumor and surrounding parenchyma during the early post-treatment period [20]. Initial findings like perilesional arterial enhancement can overlap with features of tumor progression (hence the term “pseudoprogression”) and confound treatment response assessment [23,25].

Persistent mass-like enhancement in radiation-treated HCCs could be due to the mechanism of action of radiation-induced cell death. Unlike non-radiation therapy, which induces immediate cell death from coagulation necrosis or ischemia, radiation changes can induce (i) immediate cell death, or (ii) cellular senescence [26,27]. Radiation-induced senescence is a form of proliferative cell death in which the cells are still metabolically active and maintain vascular perfusion [28] (thereby the phenomenon of enhancement), but lose their ability to proliferate and are irreversibly locked in the state of G1 phase [27]. It is believed that the aim of cell proliferative arrest via blockade in the G1 phase is to prevent DNA replication initiation in damaged cells [27,29]. In normal cells, Cyclin/Cyclin-dependent kinases (CDK) complexes drive G1/S cell-cycle progression through phosphorylation of the retinoblastoma tumor suppressor protein (RB) that decides G1 growth arrest versus proliferation. In senescence, increased expression of CDK inhibitors such as p21 and p16 results in continuous activation of RB, which sequesters E2F transcription factors and inhibits cell cycle progression. In the state of senescence, apoptosis is suppressed [29] and cell death is a slow, albeit eventual process; therefore the treated tumor can continue to display APHE after radiation therapy [26].

## 5. New Guidelines on Treatment Response Assessment for Radiation-Based Treatment

In 2024, the LI-RADS Steering Committee released an updated set of guidelines for LI-RADS TRA which addressed some limitations of the 2017 version to improve diagnostic confidence and performance, in particular, the challenges after radiation-based treatments. In view of the different imaging findings after radiation and non-radiation-based treatment, a separate algorithm for radiation-based treatment was created [6]. Currently, LI-RADS TRA is the only set of guidelines that has an algorithm which addresses radiation-based treatment response separately from non-radiation-based treatment. It was recognized that the application of the 2017 TRA after radiation treatment might cause false-positive assessment of post-treatment viability in successfully treated tumors with persistent enhancement and lead to unnecessary re-treatment. Under the new 2024 TRA guidelines for radiation-based treatment, the criteria for viable tumor were modified. Viable tumor is now defined as the presence of mass-like enhancement within the tumor or along its margin, which is new or increased in size after treatment [6]. To incorporate the concept of temporal change to reflect the delayed response typically seen after radiation treatment, a new category of “non-progressing” has replaced the previous category of “equivocal” [6,30]. This category includes mass-like enhancement in the treated tumor or along its margin that is stable or smaller in size over time after treatment [6]. The 2024 TRA guidelines also include new ancillary features of restricted diffusion and mild-moderate T2-hyperintensity. If one or both ancillary features are new or increased over time after treatment in an area of persistent mass-like enhancement, the category can be upgraded from non-progressing to viable at the reader’s discretion [6]. Another unique feature of the LI-RADS TRA guidelines is that assessment is done on a lesion-level, whereas the other major liver-specific guidelines necessitate a patient-level assessment. (A comparison of LI-RADS TRA against EASL and mRECIST treatment response guidelines is provided in Table 1).

## 6. Discussion

### 6.1. Implications for Management Plans

In view of the atypical imaging findings after radiation-based treatment due to senescence, a watch-and-wait approach is preferred despite the tumor still harboring pathologically viable tumor cells after treatment, as these viable tumor cells will continue to develop necrosis over time. In one recent study, 45% of tumors treated with SBRT and which displayed persistent enhancement, were completely necrotic on explant tissues. Furthermore, in patients with a longer time to transplantation post-SBRT, the treated tumors demonstrated greater loss of APHE (odds ratio 0.68 [95% CI: 0.45–0.91]) and a greater degree of necrosis (odds ratio 0.2 [95% CI: 0.04–0.79]) [34]. Similarly, Riaz et al. evaluated the degree of tumor necrosis on explant pathology in post-TARE HCCs and found complete pathologic necrosis in 61% of the tumors, even though there could be residual enhancement on imaging pre-transplant [19,35]. In Riaz’s study, tumors treated with TARE showed increased pathologic necrosis over time, suggesting delayed tumor death [19].

A more recent study by Wei et al., based on the LI-RADS TRA 2024 guidelines, showed that a significant proportion (61.3%) of initially non-progressing tumors (evaluated at 3–6 months) were assessed to be non-viable by 9 months [36]. Similar findings were presented in another recent study by Kim et al., which showed that the 2024 TRA guidelines effectively reflected a typical delayed response pattern after radiation treatment, with a gradual decrease in non-progressing tumors over time along with a shift to non-viable disease [30]. These corroborated the earlier findings by Stocker et al., which also reflected a delayed response after TARE, with imaging findings of viable tumor on early follow-up MRI which subsequently resolved [7]. Another study by Chiu et al. assessed the short-term outcomes associated with the LI-RADS TRA 2024 categories. Overall survival (OS) was significantly worse for patients with an initial assessment of viable than for those initially assessed as non-progressing, and most lesions initially assessed as viable remained unchanged on follow-up examination [37]. However, in contrast to the findings by Kim et al. and Wei et al., which reflected a gradual decrease in non-progressing tumors over time as they became non-viable, Chiu reported that the majority of tumors initially categorized as non-progressing (65.6%) remained unchanged in the next examination. This difference could be attributed to the different lengths of follow-up period, with Kim et al. and Wei et al. having longer follow-up periods (median of 26.7 months and 26.5 months, respectively), whereas that of Chiu et al. was shorter (median of 10 months) [30,36,37].

The above findings suggest that in non-progressing lesions, patients should not immediately undergo retreatment or salvage therapy, which could potentially cause more harm. If TARE were intended as neoadjuvant therapy before transplant or resection [4,38], a classification of “non-progressing” should perhaps not detract from the goal of transplant or resection upon successful downstaging. A non-progressing lesion should ideally be followed up with imaging surveillance, and any retreatment or salvage therapy should only be done after a thorough multidisciplinary discussion [23,26]. On the other hand, the findings by Chiu et al. seemed to support a more aggressive approach with repeat or alternate therapies for lesions categorized as viable on the first post-treatment imaging, in view of this category’s worse survival and low likelihood of improvement without further treatment [37].

### 6.2. Treatment Response Assessment Guidelines for Radiation-Based Treatment—Going Forward

Among the major HCC-specific treatment response guidelines, LI-RADS TRA is the only set of guidelines that takes into consideration the atypical imaging findings after radiation-based treatment. The other HCC-specific treatment response guidelines are limited in their ability to accurately assess response after radiation-based treatment due to the persistence of APHE in the early post-treatment period [35].

However, LI-RADS TRA is a relatively new algorithm based on expert opinion, and thus, there is a need for validation studies to support its use clinically. More studies on the performance of TRA categories to predict outcomes data, such as overall survival, time to progression, and disease-free survival, are required [28]. This shortfall is more acute with radiation-based treatment. For example, there were very few studies that evaluated the diagnostic performance of LI-RADS TRA in assessing HCC viability after SBRT and TARE using radiological-pathological correlation [34,39,40]. These studies shared similar limitations: single-centre retrospective studies with small patient numbers. In addition, the studies had inherent selection bias in that the patients included in the study underwent explantation.

Another major limitation of the 2024 LI-RADS TRA is that the ancillary features include only T2-weighted signal abnormality and diffusion restriction; other potential features, such as hepatobiliary phase (HBP) hypointensity, have not been included due to limited data. Further studies on the use of HBP hypointensity in treatment response after radiation-based treatment are needed to assess its utility for inclusion into the algorithm.

There have been very few studies which compared the outcome or accuracy across modalities in treatment response assessment after radiation-based treatment of HCC, and that is one further limitation. A study by Wei et al., based on the 2024 LI-RADS Radiation TRA, reported that there was moderate (kappa = 0.46) inter-observer agreement for CT, but almost perfect (kappa = 0.85) inter-observer agreement for MRI in LI-RADS TRA category assignment at 3–6 months [36]. In addition, MRI demonstrated superior inter-observer agreement over CT in identifying various findings such as mass-like enhancement and smooth peri-lesional enhancement. The advantages of MRI over CT are multifactorial, including its superior soft tissue contrast resolution which enhances the detection of residual tumors, and the inclusion of non-dynamic imaging sequences such as T2-weighted imaging, DWI and HBP imaging which aid in distinguishing viable tumor from inflammatory changes. Furthermore, subtraction imaging reduces the confounding effects of intra-lesional haemorrhage and calcifications [36]. Apart from the reliance on dynamic post-contrast enhancement appearances, there have also been studies that investigated diffusion restriction as a stand-alone feature for response assessment after radiation-based treatment. Furthermore, there has been increasing interest in the use of radiomics to aid treatment response assessment. Restricted diffusion and radiomics will be discussed in more detail in the following paragraphs.

### 6.3. Use of Diffusion Weighted Imaging (DWI) for Treatment Response Assessment

The effectiveness of DWI and apparent diffusion coefficient (ADC) maps in early treatment response after TARE has been evaluated in several small studies [41,42,43]. Their findings suggested that ADC values can detect increased water diffusion caused by post-TARE tissue changes earlier (around 1 month post-treatment) than conventional imaging criteria, which are usually only effective from around 3 months onwards [41,42,43]. Their findings raise the potential for DWI to serve as an early biomarker of treatment response post-TARE before changes in tumor size and morphological characteristics manifest. Viable tumors have high cellularity and cell membranes, which restrict the movement of water molecules, resulting in low ADC. Cellular necrosis post-treatment increases membranous permeability, which increases the water molecules’ freedom of movement and thereby increases ADC [42].

A recent meta-analysis evaluated the diagnostic performance of DWI/ADC for early post-TARE treatment response. The pooled sensitivity and specificity were 0.90 (95% CI: 0.75–0.96) and 0.81 (95% CI: 0.58–0.92) with DOR 45.4 (95% CI: 10.2–132). The area under the summary receiver-operating characteristic curve (AUROC) was 0.919 (95% CI: 0.708–0.924). The findings suggested that restricted diffusion had high diagnostic accuracy in early response assessment after TARE, and supported the inclusion of restricted diffusion as an ancillary criterion in LI-RADS TRA 2024 for radiation treatment [44]. Nonetheless, there are also challenges with the use of DWI to evaluate tumor viability, which can result in false positives or false negatives. Intralesional haemorrhage may cause diffusion restriction and result in a false positive finding of residual tumor. A cirrhotic liver by itself also shows increased diffusion restriction, which results in reduced HCC conspicuity on DWI. In addition, the intrinsic nature of echoplanar sequences used in DWI and limited spatial resolution can affect the diagnostic accuracy of lesion assessment [45].

### 6.4. Future Directions: Use of Radiomics to Predict Treatment Response

Radiomics involves the conversion of images to higher-dimensional data, followed by mining of this data, and provides an immense supply of imaging biomarkers that can aid treatment response assessment. Histogram analysis (first-order radiomics) is a new approach to quantify tumor heterogeneity using routine MRI data [46].

When applied to DWI imaging, histograms derived from voxel-by-voxel ADC values calculation are generated using volumetric analysis [18]. Histogram analysis uses a mathematical approach to evaluate intra-voxel gray-level intensity variations and intratumoral heterogeneity. This is superior to using mean ADC values from a single slice, which does not account for underlying tumor heterogeneity [46]. It has been postulated that post-TARE intralesional heterogeneity occurs due to non-uniform distribution of microspheres, thus volumetric ADC measurements provide more complete tumoral delineation than uni- or bi-dimensional measurements [18]. Several studies have assessed the feasibility of volumetric ADC measurements to detect early treatment response after TARE [18,46]. A study by Gordic et al. showed that changes in several volumetric ADC (ΔvADC) histogram parameters at 6 weeks post-treatment, such as ΔvADC median and ΔvADC max, were significantly different between patients with partial response/complete response versus those with stable disease/progressive disease. The ΔvADC mean at 6 weeks was also an independent predictor of complete response at 6 months [46]. Another study by Vouche et al. showed that mean ADC and ADC standard deviation (STD) changed as early as 1 month after TARE. ADC STD percentage change was significantly greater in responding lesions than in non-responding lesions at 1 month and 3 months [18].

Several studies have also evaluated the performance of radiomics derived from contrast-enhanced MRI images in post-Y90 treatment response assessment. Various predictive models using machine-learning (ML) techniques such as Logistic Regression (LR), K-Nearest Neighbours (KNN) Classification, and Support Vector Machine (SVM) models, have been applied on radiomics approaches to assess their efficacy in predicting treatment response after radiation-based treatment [47,48]. Aujay et al. identified four radiomic features (long run emphasis, minor axis length, surface area, and gray level nonuniformity on arterial phase images) that could distinguish between early (4 weeks) responders and non-responders, with better sensitivity and specificity compared with traditional imaging criteria. Long run emphasis was the best parameter for predicting early response, with 100% sensitivity (95% CI: 68−100) and 100% specificity (95% CI: 78−100) [49]. Marinelli et al. assessed the accuracy of an ML approach based on MRI radiomic quantification (shape, first-order histogram, and custom signal intensity-based radiomic features) obtained before TARE and post-TARE for the prediction of early treatment response. Their findings showed that ML modeling of radiomic data combining baseline and early follow-up MR imaging could predict early treatment response with AUROC of 0.89 (95% CI: 0.77–1.00) [48]. A recent meta-analysis of radiomic features extracted from pre- and post-TARE imaging evaluated the efficacy of radiomic models in the prediction of TARE response. Notwithstanding the heterogeneity in methodologic quality of their selected studies, the radiomic models had a pooled sensitivity of 83.5% (95% CI: 76–89%) and a pooled specificity of 86.7% (95% CI: 78–92%) in predicting complete or partial response [50].

Deep Learning (DL) is a subset of ML based on artificial neural networks (ANNs), which are modeled after the biological networks of neurons in the human brain and can process vast amounts of data through multiple layers of artificial neurons. DL models can automatically discover patterns in raw data, whereas other ML techniques need to be provided with a set of features beforehand [51]. Convoluted neural networks (CNNs) are specialized types of ANNs based on convolutional layers of neural networks, which are designed for processing digital images. CNNs can capture spatial hierarchies and patterns within images, which enables more robust feature detection and classification [52].

The application of ML/DL techniques to radiomics is intuitively beneficial to the detection of subtle patterns that are beyond the capability of human visual perception [52]. However, currently, there have only been a small handful of studies that evaluated ML techniques for treatment response assessment after radiation-based treatment [47,48]. There has not been any study that evaluated the application of DL techniques in response assessment after radiation-based treatment, and this is an area of potential development.

Despite the potential of radiomics in predicting early post-TARE response, numerous challenges exist. Standardised thresholds for indicating response are currently lacking. This limits the application of radiomics in clinical practice. Volumetric analysis requires dedicated software to ensure volumetric delineation accuracy, as well as technical expertise. Further refinements in automated segmentation, motion correction, deformable co-registration, and subtraction reconstruction would enhance existing software. Improvements in DWI spatial resolution could allow the use of iso-voxel ADC maps that would improve ADC measurements [18]. Currently, there is no suitable software for volumetric assessment and radiomics analysis that is available for routine clinical usage. With the development of automated and semi-automated segmentation tools and the standardization of radiomic features [49] (the Imaging Biomarker Standardization Initiative) [53], radiomics could become a viable clinical tool to evaluate early post-TARE treatment response [49].

## 7. Conclusions

The growing role of radiation-based treatment (TARE, SBRT) for HCC requires an understanding of the underlying biological mechanism behind radiation-induced cellular senescence for accurate post-treatment imaging interpretation. Post-imaging MRI findings after radiation-based treatment are different from those after non-radiation-based treatment, with persistent enhancement and delayed tumor shrinkage confounding the application of conventional treatment response criteria. Changes to existing treatment response assessment guidelines need to be optimized to take into account the different imaging findings after TARE and SBRT; inclusion of functional biomarkers and temporal characteristics can increase the accuracy in post-treatment imaging interpretation.

## Figures and Tables

**Table 1:** Comparison of HCC-specific treatment response criteria.

	LI-RADS TRA [1,6,26,31]		EASL [9,32,33]	mRECIST [10,32,33]
	Radiation	Non-Radiation		
Criteria	Non-viable: No mass-like enhancement present Non-progressing: Mass-like enhancement that is stable or decreased in size over time after treatment Viable: Mass-like enhancement which is new or increased in size over time after treatment	Non-viable: No mass-like enhancement present Equivocal: Uncertainty about mass-like enhancement (presence or morphology) Viable: Presence of mass-like enhancement (any degree, any phase)	^∆^CR: Disappearance of all known disease and no new lesions determined by two observations not less than 4 weeks apart ^α^PR: >50% reduction in total tumor load of all measurable lesions determined by two observations not less than 4 weeks apart ^β^SD: Does not qualify for CR, PR, or PD ^γ^PD: >25% increase in size of one or more lesions or appearance of new lesions	^∆^CR: Disappearance of intratumoral arterial enhancement in all target lesions ^α^PR: >30% decrease in the sum of diameters of viable target lesions (arterial enhancing), taking as reference the baseline sum of the diameters of target lesions ^β^SD: Does not qualify for CR, PR, or PD ^γ^PD: >20% increase in the sum of the diameters of viable (arterial enhancing) target lesions, taking as reference the smallest sum of the diameters of viable (arterial enhancing) target lesions since treatment started
Feature evaluated	Presence of mass-like enhancement (any phase) Ancillary features of DWI and T2 hyperintensity (optional)		Enhancing component in the arterial or venous phases	Enhancing component in the arterial phase
Lesions	Individual lesions		Systems Approach	Systems Approach
Measurement	Uni-dimensional		Bi-dimensional	Uni-dimensional

^∆^CR: complete response; ^α^PR: partial response; ^β^SD: stable disease; ^γ^PD: progressive disease.

## Data Availability

Not applicable.

## Abbreviations

The following abbreviations are used in this manuscript:

MRI	Magnetic Resonance Imaging
HCC	Hepatocellular carcinoma
SBRT	Stereotactic body radiotherapy
TARE	Transarterial radioembolization
LRT	Locoregional treatment
WHO	World Health Organization
EASL	European Association for the Study of the Liver
RECIST	Response Criteria in Solid Tumors
mRECIST	Modified RECIST
LI-RADS	Liver Imaging and Reporting Data System
LR-TR	LI-RADS Treatment Response
APHE	Arterial phase hyperenhancement
DWI	Diffusion Weighted Imaging
ADC	Apparent Diffusion Coefficient
PET/CT	Positron Emission Tomography/Computed Tomography
FDG	Fluorodeoxyglucose
BCLC	Barcelona Clinic Liver Cancer
SUV	Standardized Uptake Value
